# Pomegranate Heart Rot Caused by *Alternaria alternata*, an Emerging Disease in Algeria

**DOI:** 10.3390/jof12030209

**Published:** 2026-03-14

**Authors:** Nesma Abdessemed, Ali Kerroum, Sabri Ala Eddine Zaidat, Brahim Beladis, Ihssan Cherief, Rossana Parlascino, Mario Riolo, Antonella Pane, Santa Olga Cacciola

**Affiliations:** 1 Département des Sciences Agronomiques, Faculté des Sciences de la Nature et de la Vie, Université Morsli Abdellah, Tipaza 42000, Algeria; abdessemed.nesma@cu-tipaza.dz (N.A.); innaihssan@gmail.com (I.C.); 2 Laboratoire de Phytopathologie et Biologie Moléculaire, Ecole Nationale Supérieure d’Agronomie (ENSA, ex. INA), Alger 16200, Algeria; ali.kerroum@edu.ensa.dz; 3 Department of Agricultural and Veterinary Sciences, Faculty of Natural and Life Sciences, Ziane Achour University, Djelfa 17000, Algeria; zaidatsabri@hotmail.com; 4 Department of Soil, Plant and Food Sciences, University of Bari Aldo Moro, 70126 Bari, Italy; 5 Laboratoire Bioressources Sahariennes: Préservation et Valorisation, Département des Sciences Agronomiques, Université Kasdi Merbah, BP 511 Route de Ghardaïa, Ouargla 30000, Algeria; beladisbr@gmail.com; 6 Department of Agriculture, Food and Environment (Di3A), University of Catania, Via Santa Sofia 100, 95123 Catania, Italy; rossana.parlascino@phd.unict.it (R.P.); antonella.pane@unict.it (A.P.); olga.cacciola@unict.it (S.O.C.)

**Keywords:** *Alternaria alternata*, *Punica granatum*, heart rot, black heart, multilocus phylogeny, pathogenicity, cultivar susceptibility

## Abstract

Pomegranate heart rot (black heart) was observed in several pomegranate-growing areas of Algeria. From 2022 to 2025, surveys were conducted across 15 provinces (20 localities), and a total of 85 fruits (symptomatic and asymptomatic) were collected. Fruits were cut transversely to assess internal symptoms, ranging from early aril browning to dry black rot. Thirty *Alternaria* isolates were obtained and grouped into four morphotypes based on colony and conidial morphological traits. A subset of 18 isolates was analysed by multilocus phylogeny (ITS, EF-1α, GAPDH and OPA10-2); all analysed isolates clustered within the *Alternaria alternata* species complex, in the clade including the ex-type strain CBS 916.96. Fruit pathogenicity tests with Algerian isolate GA reproduced typical internal heart rot symptoms, and the pathogen was consistently re-isolated from symptomatic tissues. In fruit inoculations with isolate GA, cultivars differed in susceptibility, with mean disease severities of 94%, 62% and 9.5% in ‘Taferrante’, ‘Ikhessène’ and ‘Kares’, respectively, expressed as the percentage of the fruit section presenting rot symptoms. Detached leaf assays indicated isolate-dependent differences in aggressiveness, and ‘Kares’ showed the lowest susceptibility. Overall, the results confirm that *A. alternata* is the causal agent of pomegranate heart rot in Algeria and provide baseline information for disease diagnosis and management.

## 1. Introduction

Pomegranate (*Punica granatum* L.) is a plant native to Central Asia with a size ranging from a dwarf shrub to a tree. It has been cultivated for fruit production since ancient times. In recent years, its cultivation as a commercial fruit crop has expanded considerably in several countries, particularly in those with Mediterranean-type climates [1,2]. Pomegranate is one of the main fruit crops grown in arid regions of both the eastern and western hemispheres [3,4]. The pomegranate fruit is cultivated for its juicy grains, named arils [2,5]. It is consumed and marketed prevalently as a whole fresh fruit or extracted arils. In recent years, there has been an increasing demand for its industrially processed products, such as juice, syrup (grenadine), teas, seed oil and others [2,6,7]. Pomegranate has received considerable attention for its health benefits [8,9] due to its richness in nutrients, bioactive compounds (minerals, flavonoids and vitamins) and antioxidants [10,11,12,13]. Moreover, pomegranate peel extracts possess strong, broad-spectrum antifungal activity, and their application in agriculture as natural fungicides has been proposed [14,15]. Precise and updated estimates of global pomegranate production are not available; however, this fruit crop is widely cultivated in many countries [5,16]. According to data provided by diverse sources, the total global area devoted to pomegranate cultivation is well over 300,000 ha, and global pomegranate production is estimated to be over 3 million tonnes, of which over 76% are collectively produced by six countries (India, China, Iran, Turkey, Afghanistan and the USA) [17]. Algeria ranks among the top 10 pomegranate producers worldwide, with an area of 25,707 hectares and an annual production of around 104,700 tonnes [17]. Fungal diseases are a major constraint affecting pomegranate production worldwide [18,19,20]. The most common fungi and oomycetes infecting pomegranate fruit include *Alternaria alternata* and *A. arborescens*, *Aspergillus niger*, *Botrytis cinerea*, *Botryosphaeria dothidea*, *Colletotrichum gloeosporioides* and *C. acutatum*, *Cercospora punicae*, *Coniella granati*, *Neofusicoccum parvum*, *Penicillium implicatum*, *Pestalotiopsis versicolor* and *Phytophthora palmivora* [21,22,23,24,25,26,27,28]. During the last two decades, with the expansion of commercial pomegranate cultivation, the intensification of cropping systems and the introduction of new pomegranate cultivars many producing countries in the Mediterranean region have experienced the emergence of a fruit disease called black heart or heart rot, caused by *A. alternata*, alone or in association with other *Alternaria* species [22,23,28]. This disease has been reported in several countries, including Albania, Cyprus, Egypt, Greece, India, Italy, Iran, Israel, Spain, the United States (California) and Turkey [6,19,28,29,30,31,32,33,34,35]. It consists of an internal brown-to-black fruit decay affecting the arils, often confined to only some fruit compartments and starting from the calyx area, while the rind remains firm. *Alternaria* species infect the fruit in the field during flowering, remaining latent until favourable growing conditions occur [23,35]. Although the incidence of heart rot is relatively low, the presence of an internal rot reduces the market price of pomegranate due to the difficulty of detecting infected fruit on the basis of external symptoms [6,31]. Moreover, the *Alternaria* species responsible for heart rot produce mycotoxins that may contaminate the juice [6,22]. *Alternaria alternata* and *A. arborescens* are also responsible for Alternaria black spot, a pomegranate leaf and fruit disease distinct from heart rot [24,36,37]. However, it has not been verified if *Alternaria* isolates associated with heart rot can also infect leaves. An internal rot of pomegranate fruits with symptoms partly resembling those of heart rot incited by the *Alternaria* species is caused by *Aspergillus* spp. [28]. In recent years, symptoms of heart rot have been observed in pomegranate fruits harvested from orchards in several regions of Algeria. The aims of this study were: (i) to identify the etiological agent of heart rot of pomegranate fruits in Algeria; (ii) to characterize the *Alternaria* isolates recovered from pomegranate fruits with heart rot symptoms in Algeria; (iii) to confirm the etiology of the disease; (iv) to test the susceptibility of local pomegranate cultivars to heart rot; (v) to verify the ability of isolates obtained from fruits to infect the leaves.

## 2. Materials and Methods

### 2.1. Survey of Pomegranate Orchards and Fungal Isolation

**Figure 1:**
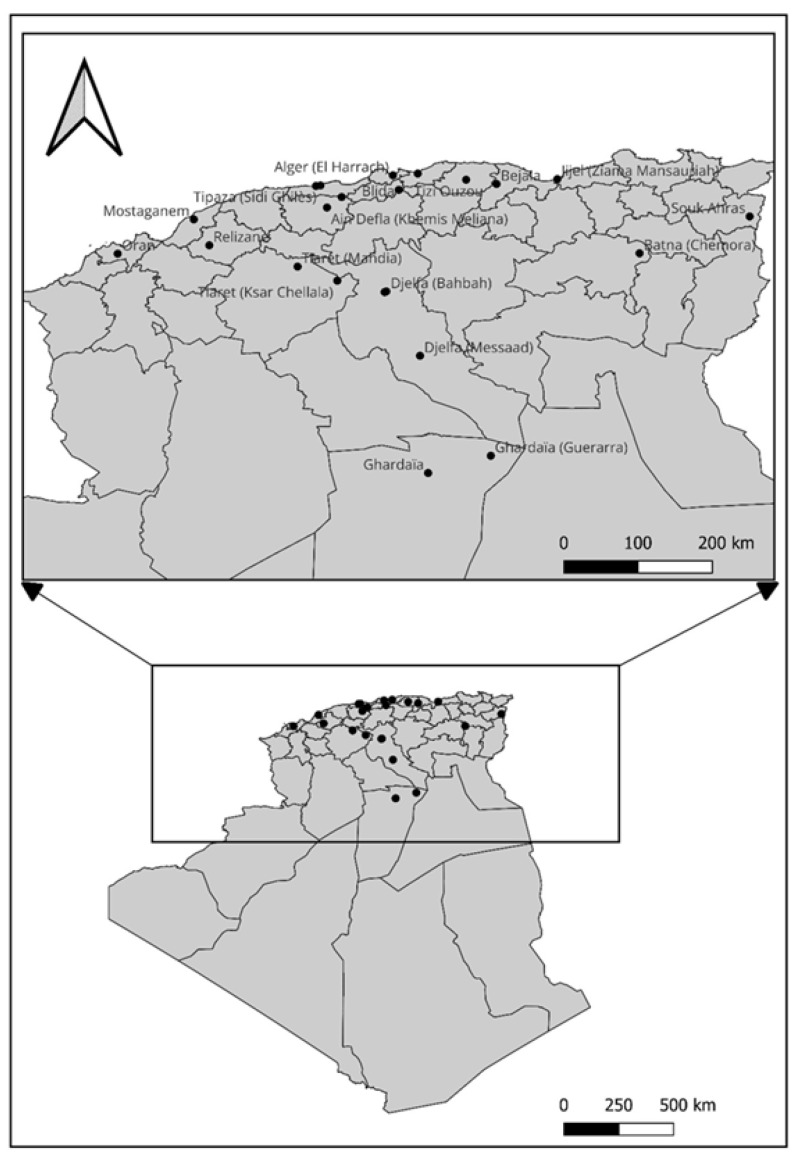
Distribution of sampling locations.

From 2022 to 2025 (in June, July and September of each year), surveys were conducted in major pomegranate-growing areas of Algeria to investigate the occurrence of heart rot and to collect fruit for fungal isolation. A total of 85 fruits were sampled from commercial orchards located in Ghardaïa (Guerrara and Ghardaïa), Djelfa (Messaad and Bahbah), Alger (El Harrach), Jijel (Ziama Mansauriah), Mostaganem (Hadjadj), Relizane (Relizane), Boumerdes (Tidjelabine), Béjaia (Chemini), Ain Defla (Boumedefaa and Khemis Meliana), Tipaza (Sidi Ghilès and Hadjret Ennous), Blida, Tiaret (Mahdia and Ksar Chellala), Tizi Ouzou (Larbaa Nath Irathen), Batna (Chemora) and Souk Ahras (Sidi Fredj) (Figure 1). During the surveys, fruits were cut transversely and inspected for internal heart rot symptoms. Field incidence was estimated visually as the proportion of inspected fruits showing internal symptoms.

Isolations were carried out from both asymptomatic and symptomatic fruits collected at the fruit-set stage and at harvest. Fruits were washed under running tap water to remove surface debris, blotted dry under sterile conditions, surface-disinfected in 3% sodium hypochlorite for 1 min, and rinsed twice in sterile distilled water (30 s each) [6,38]. Small tissue fragments (2–4 mm) were excised from the margin between healthy and rotten tissues using a sterile scalpel and plated onto Potato Dextrose Agar (PDA) [39]. Emerging colonies were subcultured on PDA. Pure cultures were obtained and deposited in the fungal collection of the Laboratory of Phytopathology and Molecular Biology, Department of Botany, ENSA (École Nationale Supérieure d’Agronomie, Algiers, Algeria).

### 2.2. Morphological Characterization of Alternaria Isolates

#### 2.2.1. Morphology of Colonies of *Alternaria* Isolates on Different Media

Isolates were grown on Potato Dextrose Agar (PDA), Potato Carrot Agar (PCA), Vegetable Juice Agar (V8A), Malt Extract Agar (MEA), Oatmeal Agar (OA), and Spezieller Nährstoffarmer Agar (SNA). Culture media were prepared in the laboratory according to standard formulations reported in previous studies [39,40]. Plates were incubated for 7 days at 22 ± 2 °C in the dark. Colony morphology was described according to standard criteria [22,41,42].

#### 2.2.2. Microscopic Features of *Alternaria* Isolates

Microscopic observations were performed on 7-day-old PDA cultures incubated at 25 ± 1 °C in the dark using the adhesive tape method [43]. Fifty conidia per isolate were randomly measured under a light microscope, recording conidial length and width, beak length, number of transverse and longitudinal septa, and number of conidia per chain [41,42]. Based on colony and conidial morphology on PDA, isolates were grouped into morphotypes, and representative isolates were selected for subsequent analyses.

### 2.3. Molecular Characterization

Isolates were grown on PDA for 7 days at 25 ± 1 °C. Mycelium from each isolate was harvested using a sterile scalpel, and genomic DNA was extracted using the PowerPlant^®^ Pro DNA Isolation Kit (MO BIO Laboratories, Inc., Carlsbad, CA, USA), following the manufacturer’s protocol. The DNA was stored at −20 °C. To characterize and determine the phylogenetic allocation of the isolates, a multilocus approach was adopted. Portions of four loci/regions were amplified and sequenced, including the internal transcribed spacer (ITS), the translation elongation factor 1-α (EF-1α), the glyceraldehyde-3-phosphate dehydrogenase gene (GAPDH), and a specific SCAR marker (OPA10-2). The primer pairs used were ITS1/ITS4 for ITS [44], EF1-728F/EF1-986R for EF-1α [45], GPD1/GPD2 for GAPDH [46], and OPA10-2R/OPA10-2L for OPA10-2 [24,47]. PCR amplifications were carried out using the GeneAmp PCR System 9700 (Applied Biosystems, Monza-Brianza, Italy). The reaction protocol comprised an initial denaturation at 94 °C for 3 min followed by 35 cycles of 94 °C for 30 s, annealing at 55 °C (ITS), 58 °C (EF-1α), 54 °C (GAPDH) or 62 °C (OPA10-2) for 30 s, and an extension at 72 °C for 30 s, with a final extension at 72 °C for 10 min. Amplicons were visualized on a 1% agarose gel, and purified products were sequenced with both forward and reverse primers by Macrogen Europe (Amsterdam, The Netherlands).

### 2.4. Evaluation of Optimal Conditions for Mycelial Growth and Sporulation

#### 2.4.1. Effect of Temperatures on the Growth of *Alternaria* Isolates

To determine the effect of temperature on mycelial growth, *Alternaria* isolates were incubated at different temperatures (10, 15, 20, 25, 30 and 35 °C) [42,48]. Mycelial explants (5 mm in diameter) were aseptically extracted from seven-day-old cultures using a sterile Pasteur pipette and placed at the centre of each Petri dish containing PDA. Plates were incubated in the dark for 7 days at the above temperatures. After incubation, colony diameter was measured (mm) using a ruler. Colony diameter was measured after 7 days of incubation. The mean daily mycelial growth rate (mm day^−1^) was calculated as:

$$Daily\;growth\;rate=\frac{\left(Colony\;diameter\;at\;day\;7-Initial\;plug\;diameter\right)}{Days\;of\;incubation}$$

where the initial plug diameter was 5 mm and incubation period was 7 days [49]. Each isolate × temperature combination included three replicate plates.

#### 2.4.2. Effect of Media on the Growth of *Alternaria* Isolates

To evaluate the effect of different culture media on mycelial growth and sporulation, the isolates were grown on PDA, PCA, V8A, MEA, OA and SNA. Mycelial agar plugs (5 mm in diameter) were aseptically removed from the growing margin of 7-day-old colonies using a tapered sterile Pasteur pipette and placed at the centre of each Petri dish. Dishes were incubated at 20 °C in the dark for 7 days. At the end of incubation, colony diameter (mm) was measured using a ruler. The growth rate was calculated using the same formula reported in Section 2.4.1. Each isolate × medium combination included three replicate plates.

Sporulation was assessed from 7-day-old colonies. Each Petri dish was flooded with 10 mL of sterile distilled water (SDW), and the colony surface was gently scraped using a curved sterile Pasteur pipette. The resulting suspension was filtered and the conidial concentration was determined using a hemocytometer (Malassez counting chamber), as described by Bessadat et al. [42].

#### 2.4.3. Pathogenicity Tests on Fruit

Fruits of three pomegranate cultivars (Taferrante, Ikhessène and Kares) were used for pathogenicity tests. In July 2023, fruits at the pre-maturity stage were inoculated with a conidial suspension (1 × 10^6^ conidia mL^−1^) prepared from isolate GA (morphotype 1), selected as a representative isolate of the *Alternaria* population recovered from symptomatic fruits following multilocus identification of a subset of isolates. Each fruit was injected with 50 µL of the suspension using a sterile syringe at two opposite points on the equatorial axis. For each cultivar, 24 fruits on three distinct trees were used, including 12 inoculated fruits and 12 control fruits (four inoculated and four control fruits per tree) injected with sterile distilled water (SDW). Inoculated and control fruits were randomly distributed around the canopy (four cardinal directions) on three trees per cultivar. Eight weeks after inoculation, fruits were harvested and transferred to the laboratory for symptom assessment. Fruits were cut transversely and the extent of internal black rot was recorded. Disease severity was expressed as the percentage of the fruit cross-sectional area affected by black rot. The total section area and symptomatic area were traced on a transparent cellophane sheet and measured using a portable leaf area meter (CI-202, CID Bio-Science, Camas, WA, USA). Reisolations were performed from symptomatic tissues and from control fruits [22].

### 2.5. Detached Leaf Assay

To evaluate the ability of *Alternaria* isolates recovered from pomegranate fruits to cause symptoms on leaves, a detached leaf assay was performed. Healthy leaves collected from the pomegranate cultivars Taferrante, Ikhessène and Kares were surface-disinfected in 1% sodium hypochlorite for 2 min, rinsed three times with sterile distilled water (SDW) and dried on sterile filter paper. Leaves were placed abaxial side up on moist filter paper in sterile glass Petri dishes (9 cm diameter) used as moist chambers (two leaves per dish). Leaves were inoculated with 20 µL drops of a conidial suspension (1 × 10^6^ conidia mL^−1^) of the isolates GA, GM1 and Tf1 (representative isolates of morphotypes 1, 2 and 4, respectively). Four drops were applied per leaf (two on each side of the midrib). Control leaves received the same volume of SDW. Plates were incubated at 20 °C for 7 days. At 7 days post-inoculation, the radius of the necrotic lesion at each inoculation site was measured, and lesion area was calculated as A = πr^2^. In each independent run, four moist chambers (two leaves per chamber) were used per cultivar × isolate combination, and the assay was repeated twice as independent experiments. The eight values of the area of necrotic lesions around the inoculation sites in the two leaves of each moist chamber were averaged. The moist chamber was considered the experimental unit for statistical analysis. To fulfil Koch’s postulates, reisolations were performed from symptomatic lesions, and isolates were identified both morphologically and molecularly [24].

### 2.6. Data Analysis

Sequences were checked and edited using FinchTV v.1.4.0 and compared with sequences available in GenBank using BLASTn on the NCBI web server (accessed on 10 January 2026). For phylogenetic inference, duplicate reference sequences were removed using ElimDupes software; alignments were generated using MUSCLE v3. Phylogenetic analyses were performed in MEGA6 using the Maximum Likelihood method with the Tamura–Nei model and 1000 bootstrap replications. A combined dataset of ITS, EF-1α, GAPDH and OPA10-2 was used for multilocus analyses.

Data from laboratory and field experiments were subjected to analysis of variance (ANOVA). One-way ANOVA was used when a single factor was tested, whereas two-way ANOVA was used for factorial experiments. Prior to analysis, residuals were checked for normality and homogeneity of variances; data were transformed when necessary to meet ANOVA assumptions, and untransformed means are reported. When significant effects were detected, means were separated using Tukey’s HSD test (*p* ≤ 0.05). All analyses were performed using STATISTICA software (v.8.5; StatSoft Inc., 2014)

## 3. Results

### 3.1. Symptoms and Incidence of Disease

**Figure 2:**
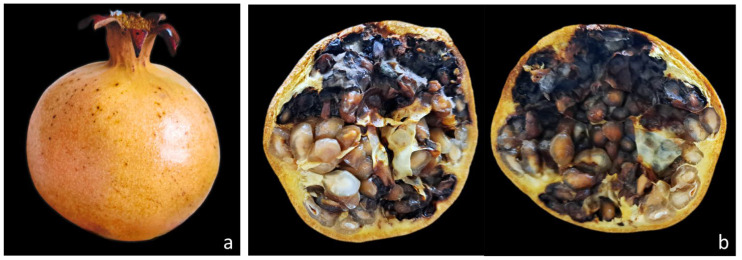
Pomegranate fruit with heart rot collected from oases in Ghardaïa district: (**a**) outside; (**b**) internal symptoms.

Mature fruits were naturally infected and collected from different regions of Algeria and showed no evident external rot symptoms. Only a few fruits showed a slight discoloration of the pericarp (Figure 2a) and a slight wrinkling of the rind corresponding to the internal rot. After cutting, fruits showed a dry black rot affecting the arils (Figure 2b). Fruits collected at the fruit-setting stage were externally symptomless but showed initial browning of the arils. Infected fruits were lighter and often slightly asymmetrical, and some showed a wavy peel corresponding to the internal rot. Premature fruit drop was frequently observed. Disease incidence, estimated by visual inspection after transverse cutting, reached approximately 20–30% in some pomegranate-growing areas, including Messâad (Djelfa) and the Ghardaïa districts.

### 3.2. Alternaria Isolates Collection

Thirty *Alternaria* isolates recovered from symptomatic pomegranate fruits were tentatively grouped into four morphotypes based on macro- and microscopic traits on PDA (Table 1). Morphotype 3 was the most frequent (12 isolates), followed by morphotypes 1 and 2 (8 isolates each), whereas morphotype 4 was represented by only two isolates.

### 3.3. Morphological and Cultural Characterization of Alternaria Isolates

#### 3.3.1. Effect of Different Media on the Colony Morphology of *Alternaria* Isolates

**Figure 3:**
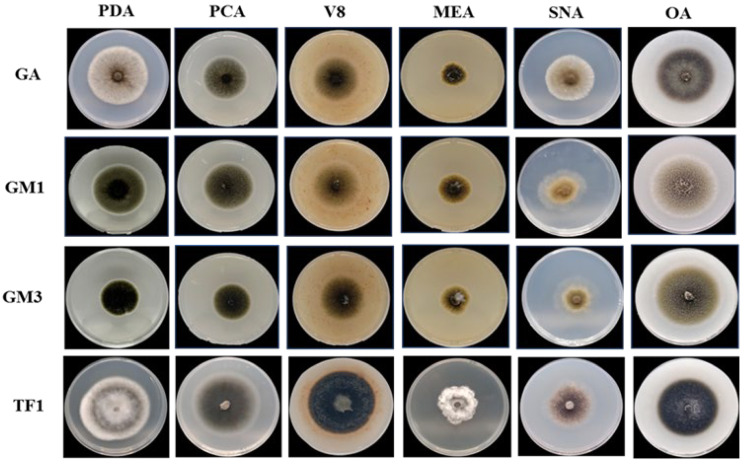
Colony morphology of representative *Alternaria* isolates GA, GM1, GM3 and Tf1 of morphotype 1, 2, 3 and 4 on different media (PDA, PCA, V8, MEA, SNA and OA).

Four representative *Alternaria* isolates, one for each morphotype, were selected for accurate characterization, namely GA (morphotype 1), GM1 (morphotype 2), GM3 (morphotype 3) and Tf1 (morphotype 4). Colony morphology was evaluated on six culture media (PDA, PCA, V8A, MEA, SNA and OA) (Figure 3). Overall, colonies ranged from grey/olive-green to dark grey and differed in margin definition, aerial mycelium development and pigmentation depending on isolate and medium. MEA consistently resulted in slower growth compared with the other media.

Isolate GA (morphotype 1) showed the highest radial growth on PDA and produced grey to olive-green colonies with a well-defined margin. On OA, colonies were flat and dark green with a lighter central area.

Isolates GM1 (morphotype 2) and GM3 (morphotype 3) showed very similar colony patterns across most media. On PDA and PCA, colonies were typically dark olive-green with a thin white margin. On SNA, they developed an olive-green central area surrounded by a hyaline peripheral zone with a loose mycelium.

Isolate Tf1 (morphotype 4) was readily distinguishable by concentric white and grey rings on PDA. On MEA, colonies were slow-growing with irregular edges and abundant aerial mycelium. On the other media, Tf1 produced dark grey colonies with well-defined margins (Figure 3).

#### 3.3.2. Microscopic Features of the Representative *Alternaria* Isolates

All four representative isolates, GA (morphotype 1), GM1 (morphotype 2), GM3 (morphotype 3) and Tf1 (morphotype 4), produced melanized, septate mycelium and primary conidiophores that were straight or slightly curved and either simple or branched, except for GA, whose conidiophores were predominantly unbranched. The conidia of GA were mainly obclavate, slightly swollen and dark brown. The conidia of GM1 and GM3 were obclavate to ellipsoidal and greyish-green to light olive-green, whereas Tf1 produced obclavate to oblong conidia that were olive-green in colour. Conidia of all isolates showed transverse and longitudinal septa and a short beak and were produced in short acropetal chains (≤5 conidia); chains were generally branched, except for GM3, which formed mainly unbranched chains. Conidial measurements (*n* = 50) and septation are reported in Table 2.

### 3.4. Phylogenetic Analyses

**Figure 4:**
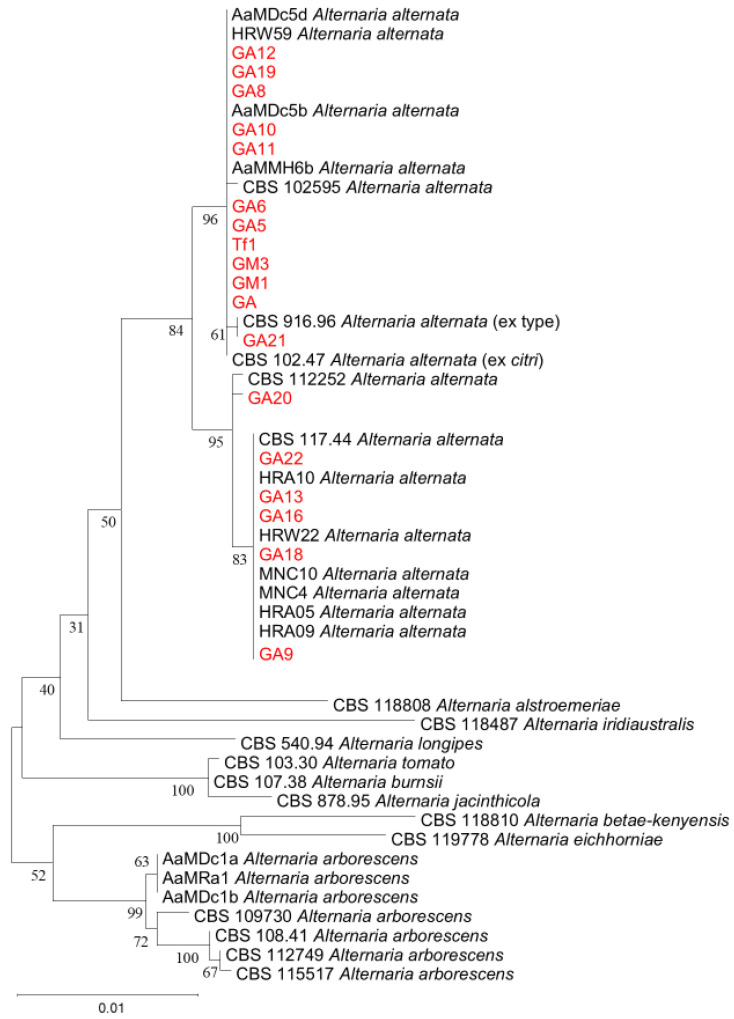
Maximum likelihood phylogenetic tree inferred from the combined dataset of ITS, EF-1α, GAPDH and OPA10-2 sequences of *Alternaria* isolates recovered from pomegranate fruits with heart rot symptoms in Algeria and reference strains from GenBank. The analysis was performed in MEGA version 11.0.13 using the Tamura–Nei model; bootstrap values (1000 replicates) are shown at the nodes. Isolates from the present study are indicated in red. *Alternaria betae-kenyensis* and *Alternaria eichhorniae* were used as outgroups. The tree with the greatest log likelihood (-3746.14) is shown.

Of the 30 isolates obtained in this study, a subset of 18 was selected for molecular analysis and included in a four-gene phylogeny based on ITS, EF-1α, GAPDH and OPA10-2 sequences, together with reference CBS strains and other reference isolates retrieved from GenBank, including isolates from pomegranate (Table S1) [6,22,24,50]. According to the phylogenetic analysis, all isolates from Algeria clustered within the *Alternaria alternata* species complex, grouping with reference strains including the *A. alternata* ex-type CBS 916.96, *A. alternata* ex *citri* CBS 102.47 and CBS 112252 (Figure 4). None of the isolates from Algeria grouped with other *Alternaria* species included in the analysis. All sequences generated in this study were deposited in GenBank; accession numbers of isolates from Algeria and reference isolates included in the phylogeny are reported in Table S1.

### 3.5. Optimal Conditions for Mycelial Growth and Sporulation of Alternaria Isolates

#### 3.5.1. Effect of Temperatures on Mycelial Growth of *Alternaria* Isolates

**Figure 5:**
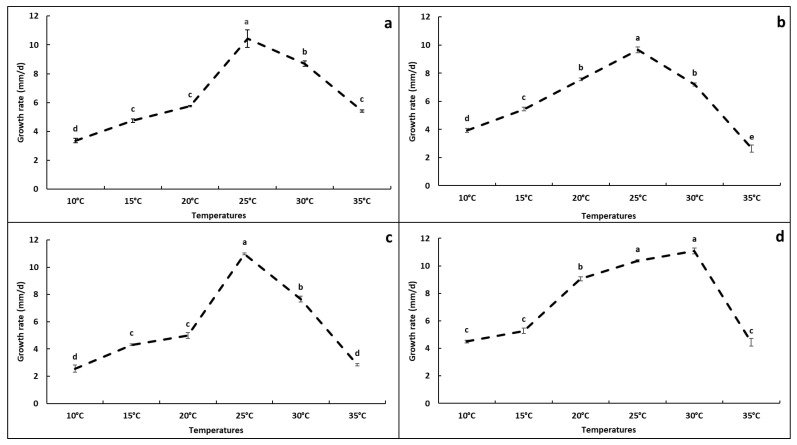
Effect of temperature on mycelial growth of *Alternaria* isolates recovered from pomegranate fruits with symptoms of heart rot in Algeria: (**a**) GA, (**b**) GM1, (**c**) GM3, (**d**) Tf1. Values are means of three replicate plates. The absence of common letters indicates significant differences among temperatures within each isolate according to Tukey’s HSD test (*p* ≤ 0.05).

Analysis of variance (ANOVA) showed a significant effect of temperature on the mycelial growth of the four representative isolates (*p* ≤ 0.05) (Figure 5). The optimal temperature for GA (morphotype 1), GM1 (morphotype 2) and GM3 (morphotype 3) was 25 °C, with growth rates of 10.44, 9.66 and 10.97 mm day^−1^, respectively. For Tf1 (morphotype 4), the highest growth rate was recorded at 25 °C (10.36 mm day^−1^), and 30 °C also supported optimal growth. At 10 °C, growth rates were 3.36, 3.94, 2.55 and 4.50 mm day^−1^ for GA, GM1, GM3 and Tf1, respectively. At 35 °C, growth was strongly reduced but not completely inhibited, with growth rates of 5.41, 2.63, 2.83 and 4.44 mm day^−1^ for GA, GM1, GM3 and Tf1, respectively (Figure 5).

#### 3.5.2. Effect of Media on Mycelial Growth and Sporulation of *Alternaria* Isolates

Analysis of variance (ANOVA) showed a significant effect of culture medium on both the mycelial growth and the sporulation of the four representative isolates (*p* ≤ 0.05) (Figure 5). Overall, MEA consistently resulted in reduced growth rates compared with the other media, whereas PDA and/or OA generally supported the highest growth.

**Figure 6:**
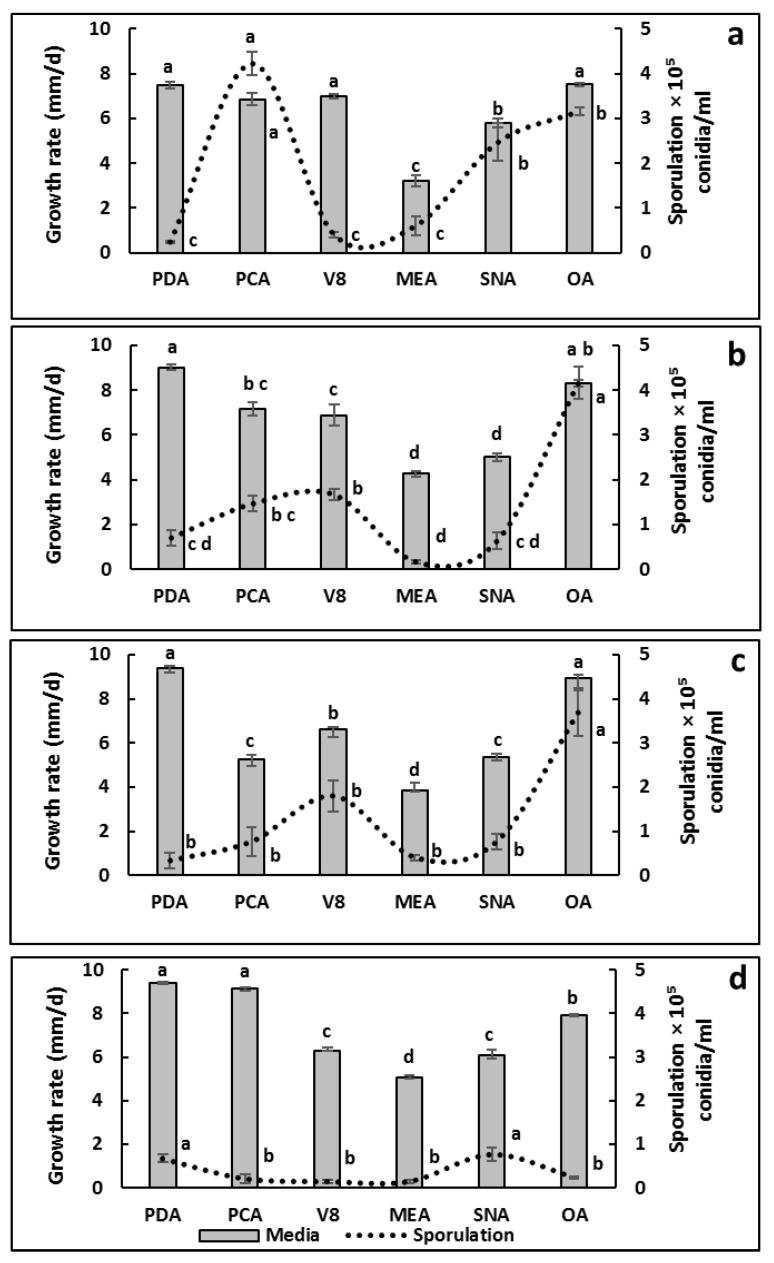
Effect of culture media on mycelial growth (bars) and sporulation (dotted line) of *Alternaria* isolates recovered from pomegranate fruits with symptoms of heart rot in Algeria: (**a**) GA, (**b**) GM1, (**c**) GM3, (**d**) Tf1. Values are means of three replicate Petri dishes. The absence of common letters indicates significant differences among media within each isolate according to Tukey’s HSD test (*p* ≤ 0.05).

For GA (morphotype 1), the highest growth rates were recorded on PDA, PCA, V8A and OA (6.86–7.53 mm day^−1^), while growth was reduced on SNA and particularly on MEA (5.77 and 3.19 mm day^−1^, respectively). Sporulation was highest on PCA (4.23 × 10^6^ conidia mL^−1^), followed by OA and SNA, whereas it was lowest on PDA, V8A and MEA (Figure 6a).

For GM1 (morphotype 2), the highest growth rate was observed on PDA (9.00 mm day^−1^), followed by OA, PCA and V8A (6.87–8.30 mm day^−1^); the lowest growth rates were recorded on MEA and SNA (4.24 and 5.00 mm day^−1^, respectively). Sporulation was highest on OA (4.17 × 10^6^ conidia mL^−1^), followed by V8A and PCA, whereas the lowest values were recorded on MEA and SNA (Figure 6b).

For GM3 (morphotype 3), the highest growth rates were recorded on PDA and OA (9.39 and 8.92 mm day^−1^, respectively), followed by V8A (6.61 mm day^−1^), whereas PCA, SNA and MEA supported lower growth (3.86–5.36 mm day^−1^). Sporulation was highest on OA (3.70 × 10^6^ conidia mL^−1^) and lower on the other media (Figure 6c).

For Tf1 (morphotype 4), the highest growth rates were obtained on PDA and PCA (9.42 and 9.17 mm day^−1^, respectively), followed by OA (7.91 mm day^−1^), while the lowest growth rate was recorded on MEA (5.05 mm day^−1^). Tf1 showed overall lower sporulation than the other isolates; the highest values were recorded on PDA and SNA (0.77 × 10^6^ and 0.66 × 10^6^ conidia mL^−1^, respectively), whereas sporulation was negligible on OA, PCA, MEA and V8A (Figure 5d).

### 3.6. Pathogenicity Tests

**Figure 7:**
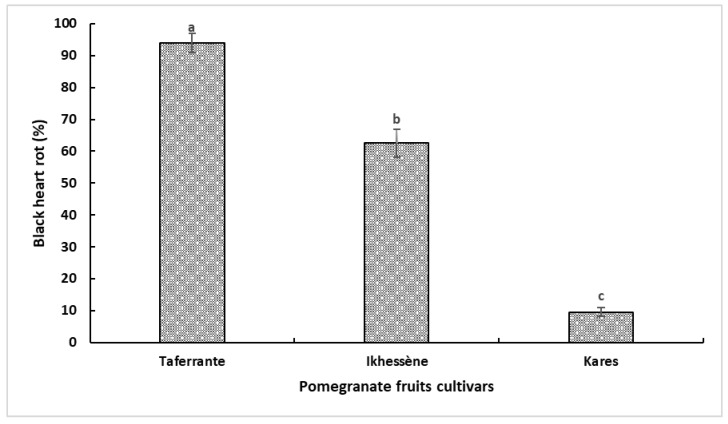
Disease severity (percentage of fruit cross-sectional area affected by black rot) in fruits of three pomegranate cultivars (Taferrante, Ikhessène and Kares) eight weeks after inoculation with *Alternaria alternata* isolate GA. Different letters indicate significant differences among media within each pomegranate cv. according to Tukey’s HSD test (*p* ≤ 0.05).

**Figure 8:**
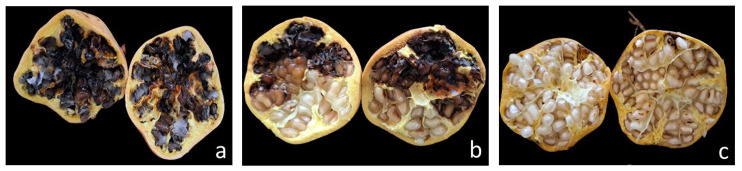
Internal symptoms of heart rot in fruits of three pomegranate cultivars eight weeks after artificial inoculation with *Alternaria alternata* isolate GA from Algeria: (**a**) cv. Taferrante; (**b**) cv. Ikhessène; (**c**) cv. Kares.

All sequenced isolates were assigned to *Alternaria alternata* in the multilocus phylogeny (Figure 4). Fruit inoculations were therefore performed using isolate GA as a representative isolate. Koch’s postulates were fulfilled. Typical heart rot symptoms were observed in the fruits of the three pomegranate cultivars (Taferrante, Ikhessène and Kares) inoculated with the conidial suspension of *Alternaria alternata* isolate GA (morphotype 1) eight weeks after injection (Figure 7 and Figure 8). Control fruits injected with sterile distilled water remained asymptomatic. *A. alternata* was consistently re-isolated from symptomatic tissues of all cultivars and showed the same colony morphology and microscopic features as the original isolate. In fruit inoculations with isolate GA, cultivars differed in susceptibility. Mean disease severity, expressed as the percentage of the fruit cross-sectional area affected by black rot, was 94% in Taferrante, 62% in Ikhessène and 9.5% in Kares (Figure 7).

### 3.7. Pathogenicity on Detached Leaves 

**Figure 9:**
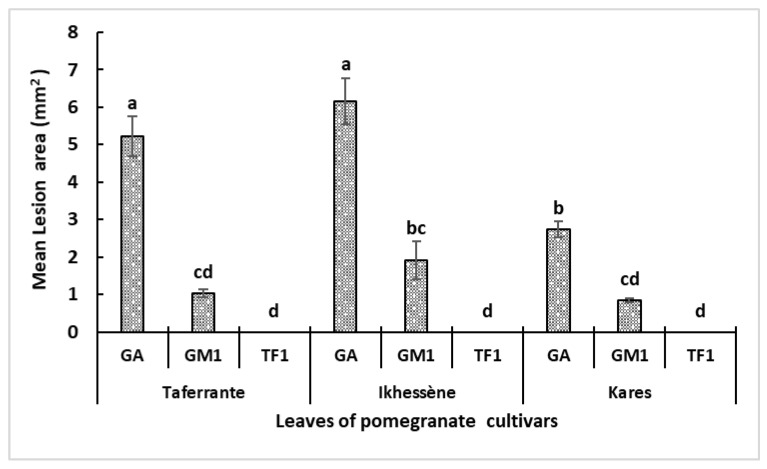
Mean lesion area (mm^2^) on detached leaves of three pomegranate cultivars (Taferrante, Ikhessène and Kares) seven days after inoculation with *Alternaria* isolates GA, GM1 and Tf1. Values are means ± SE. The absence of common letters indicates significant differences among treatments according to Tukey’s HSD test (*p* ≤ 0.05).

**Figure 10:**
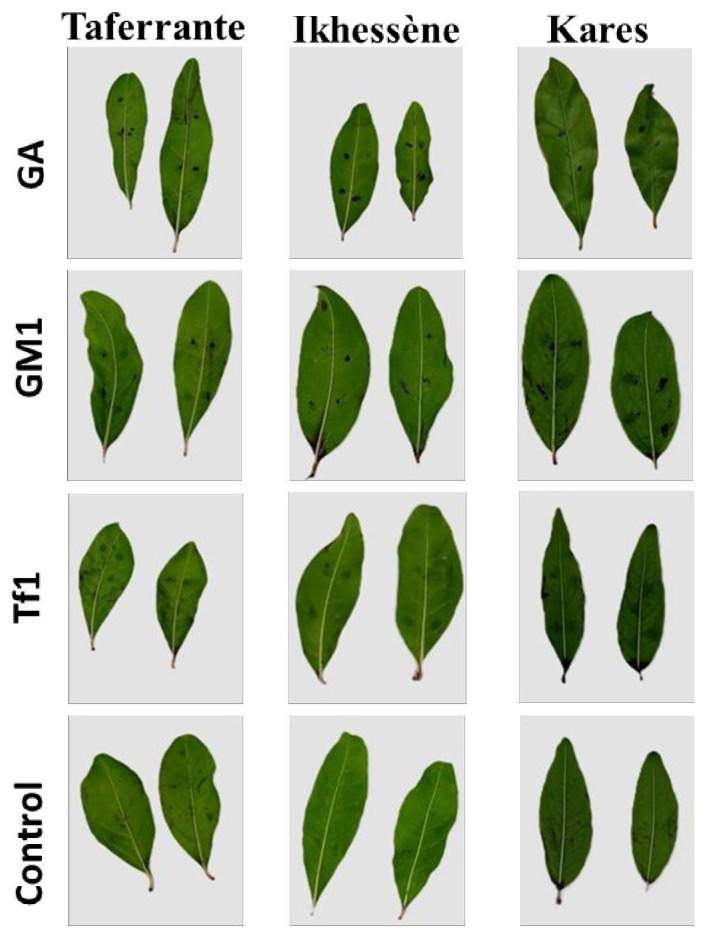
Symptoms on detached leaves of three pomegranate cultivars seven days after inoculation with conidial suspensions of *Alternaria alternata* isolates GA, GM1 and Tf1; control leaves treated with sterile distilled water (SDW) are also shown.

Two-way ANOVA showed a significant effect of isolate on lesion area on detached leaves of the three pomegranate cultivars (*p* < 0.001) (Figure 9). Isolates GA and GM1 induced necrotic lesions on leaves of all cultivars, with larger lesions caused by GA than GM1. Mean lesion area after 7 days was 5.21, 6.14 and 2.73 mm^2^ for GA on Taferrante, Ikhessène and Kares, respectively, whereas GM1 produced smaller lesions (1.03, 1.91 and 0.85 mm^2^, respectively). In contrast, Tf1 did not induce lesions under the conditions tested. Control leaves treated with sterile distilled water (SDW) remained symptomless (Figure 9 and Figure 10).

## 4. Discussion

This study reports pomegranate heart rot in Algeria and provides a characterization of the associated *Alternaria* population including symptom assessment, morphological grouping, multilocus phylogeny, pathogenicity tests, and in vitro growth and sporulation assays. Naturally infected fruits showed limited or no external symptoms, whereas internal tissues showed a dry black rot affecting the arils. This symptom pattern agrees with previous reports of heart rot (black heart) in pomegranate, where internal decay may remain unnoticed until cutting and causes postharvest losses due to ineffective visual sorting [19,22,23,26,29,31,32,34,51]. In the surveyed Algerian areas, the heart rot incidence reached 20–30% in some locations, indicating that the disease can represent a relevant constraint under local production conditions. This incidence is higher than that reported in other pomegranate-producing countries, such as the United States (California) and Italy [31,34]. Conversely, comparable field incidence levels have been reported in other production areas, probably depending on cultivar and orchard conditions [30]. These observations suggest that cultivar choice and orchard conditions may influence disease impact under local production systems.

Visual grading of intact fruit is unreliable as symptoms of heart rot are almost exclusively internal. This has motivated the development of non-destructive tools to improve detection, including imaging-based approaches [51,52,53]. These studies confirm that the internal nature of heart rot is a critical aspect of the pomegranate fruit trade and support the need to focus on orchard-based prevention rather than relying only on postharvest sorting.

Based on morphological traits, the *Alternaria* isolates recovered from pomegranate fruits in Algeria were grouped into four morphotypes, indicating phenotypic variability within the sampled population. However, multilocus phylogenetic analysis based on ITS, EF-1α, GAPDH and OPA10-2 placed all sequenced isolates within the *A. alternata* species complex and clustered them with reference strains, including the ex-type CBS 916.96. This result confirms that morphology alone is not sufficient for reliable species delimitation in small-spored *Alternaria* and supports the use of multilocus approaches for robust identification and comparability among studies [6,22,23,34,41,50]. The presence of multiple morphotypes within a single phylogenetic species is consistent with previous studies that evidenced the phenotypic plasticity of *A. alternata* populations associated with pomegranate [22,24].

Pathogenicity tests fulfilled Koch’s postulates and confirmed the etiological role of *A. alternata* in heart rot development in Algerian pomegranate orchards. Typical internal symptoms were reproduced after inoculation, and the pathogen was consistently re-isolated from symptomatic tissues. Similar approaches have been used to confirm the involvement of *Alternaria* species in pomegranate heart rot in other production areas [19,29,31,34]. In the present study, in fruit inoculations with isolate GA, the three tested Algerian pomegranate cultivars were shown to differ markedly in susceptibility to heart rot, with ‘Kares’ being significantly less susceptible than ‘Ikhémene’ and ‘Taferrante’ in artificial inoculations. A limitation of this study is that fruit pathogenicity was assessed using one representative isolate (GA). Further studies will evaluate the aggressiveness/virulence among multiple isolates to assess their potential impact on cultivar response. Consistent with this finding, differences in susceptibility to *Alternaria* isolates recovered from leaves and fruits infected by Alternaria black spot have been reported among pomegranate cultivars [24]. This aspect deserves to be studied as host genetic resistance is crucial for disease management. Although the causes predisposing to heart rot infection are not fully understood, in a screening of several pomegranate cultivars, juice pH was found to be a key factor in conditioning the germination of *A. alternata* conidia [6]. However, several lines of evidence indicate that other factors besides juice pH are involved in determining the susceptibility of pomegranate cultivars to heart rot [28]. The detached leaf assay demonstrated that *Alternaria* isolates associated with fruit heart rot can infect the leaves but highlighted variability among the isolates. The isolates GA and GM1 induced necrotic lesions on leaves of all pomegranate cultivars tested, whereas isolate Tf1 did not cause symptoms. Moreover, isolate GA caused significantly larger lesions than isolate GM1. This suggests intraspecific variability in both pathogenicity and virulence. Consistent with these findings, Tirrò et al. [24] observed variability in pathogenicity among *A. alternata* isolates associated with pomegranate Alternaria black spot.

Temperature and culture media significantly affected mycelial growth and sporulation of the *Alternaria* isolates recovered from heart-rot-affected pomegranate fruits. Cardinal temperatures for mycelial growth of isolates recovered from pomegranate fruits in Algeria are consistent with those reported for *A. alternata* by other authors [34,54]. Medium- and isolate-dependent differences in growth and sporulation observed in this study agree with previous reports [22,41,42]. These results have implications for standardizing morphological characterization of *Alternaria* isolates and for optimizing inoculum production in future pathogenicity assays.

Beyond yield losses and fruit marketability, heart rot has implications for fruit quality and food safety. *Alternaria alternata* can produce secondary metabolites and mycotoxins of concern for consumers [55]. *Alternaria* toxins have been detected in the fruits and juices of pomegranate, and technological processing steps may not be sufficient to eliminate contamination [6,56,57]. Pomegranate fruits infected by heart rot may easily contaminate the juice processing chain, as external symptoms are deceptive or, in most cases, absent [6,22]. As far as the pomegranate industry in Algeria is concerned, future studies should focus on the distribution and incidence of heart rot in diverse production areas and in relation to cropping systems, the disease epidemiology, the susceptibility of commercial cultivars and the evaluation of the risk of juice contamination by mycotoxins. Modern diagnostic methods, as recently reviewed by Parlascino et al. [58], may be a useful tool to pursue these objectives.

## 5. Conclusions

Heart rot represents an emerging issue for pomegranate production in Algeria, with potential impacts on marketability and postharvest losses. The disease may remain externally unapparent, while internal tissues develop a dry black rot affecting arils and internal membranes, limiting the effectiveness of visual sorting. The causal agent can be consistently assigned to the *Alternaria alternata* species complex using a multilocus approach, and pathogenicity assays confirm its role in internal fruit decay. Environmental conditions influence fungal growth and sporulation, with an optimum close to 25 °C and a marked reduction at 35 °C, which supports the relevance of orchard microclimate and handling conditions. Phenotypic variability among isolates is consistent with intraspecific differences and supports broader testing of isolates to better define aggressiveness. Overall, prevention strategies in Algeria should integrate orchard management, cultivar choice, and postharvest practices aimed at reducing latent infections and limiting losses along the supply chain.

## Figures and Tables

**Table 1:** List of *Alternaria* isolates recovered from pomegranate fruits in Algeria, along with isolate code, morphotype assignment, cultivar and geographic origin. The column “Sequenced” indicates isolates selected for multilocus sequencing (+) or not sequenced (−).

Isolate Code	Morphotype	Cultivar	Origin	Sequenced
GA	1	Taferrante	Ghardaïa (Ghardaïa)	+
GA10	1	Melycé	Jijel (Ziama Mansauriah)	+
GA12	1	ND	Tizi Ouzou (Larbaa Nath Irathen)	+
GA13	1	ND	Tizi Ouzou (Larbaa Nath Irathen)	+
GA16	1	ND	Tizi Ouzou (Larbaa Nath Irathen)	+
GA18	1	Maazi	Jijel (Ziama Mansauriah)	+
GA5	1	ND	Alger (El Harrach)	+
GA7	1	Snin laaloudj tounsi	Djelfa (Messaad)	−
GA17	2	Snin laaloudj tounsi	Djelfa (Messaad)	−
GA22	2	ND	Tizi Ouzou (Larbaa Nath Irathen)	+
GA23	2	Gabsi	Batna (Chemora)	−
GA24	2	Ikhessène	Relizane (Relizane)	−
GA29	2	Snin Laaloudj tounsi	Djelfa (Messaad)	−
GA30	2	ND	Souk Ahras (Sidi Fredj)	−
GA6	2	ND	Alger (El Harrach)	+
GM1	2	Khadraya	Djelfa (Messaad)	+
GA11	3	ND	Tiaret (Mahdia)	+
GA14	3	ND	Tizi Ouzou (Larbaa Nath Irathen)	−
GA15	3	ND	Tizi Ouzou (Larbaa Nath Irathen)	−
GA19	3	ND	Tiaret (Mahdia)	+
GA20	3	ND	Tiaret (Mahdia)	+
GA21	3	ND	Tizi Ouzou (Larbaa Nath Irathen)	+
GA25	3	ND	Batna (Chemora)	−
GA26	3	ND	Tiaret (Ksar Chellala)	−
GA27	3	ND	Bejaia (Chemini)	−
GA28	3	ND	Batna (Chemora)	−
GA8	3	ND	Ghardaïa (Guerarra)	+
GM3	3	Khadraya	Djelfa (Messaad)	+
GA9	4	ND	Djelfa (Bahbah)	+
Tf1	4	Taferrante	Ghardaïa (Ghardaïa)	+

ND: Not determined.

**Table 2:** Microscopic features of representative *Alternaria* isolates recovered from pomegranate fruits with symptoms of heart rot in Algeria and reference isolates from other pomegranate-growing countries.

*A. alternata* Isolates from Algeria and Reference Isolates	Conidial Size (Length × Width, μm)	Beak Length (µm)	Septa *	References
GA	15–46.25 × 8.75–16.25	0–6.25	0–2 long.; 1–4 trans.	Present study
GM1	12.5–35 × 5–12.5	0–7.50	0–2 long.; 1–4 trans.	Present study
GM3	10–32.5 × 5–13.75	0–6.25	0–2 long.; 1–4 trans.	Present study
Tf1	15–60 × 6.25–16.25	0–12.5	0–5 long.; 2–6 trans.	Present study
*A. alternata*	9–30 × 5–12	/	0–5 long.; 1–7 trans.	[31]
*A. alternata*	10–21 × 4–10	**/**	**/**	[29]
*A. alternata*	11–27 × 5–8	1.9–3.70	0–2 long.; 2–5 trans.	[19]
*A. alternata*	8–28 × 4–12	/	0–2 long.; 1–6 trans.	[26]

* trans.: transverse septa; long.: longitudinal septa; /: not reported.

## Data Availability

The original contributions presented in this study are included in the article. Further inquiries can be directed to the corresponding author.

## Supplementary Materials

The following supporting information can be downloaded at: https://www.mdpi.com/article/10.3390/jof12030209/s1, Table S1: GenBank accession numbers of ITS, EF-1α, GAPDH and OPA10-2 sequences of *Alternaria* isolates from this study and reference isolates of different host and country origins included in the phylogenetic analyses.
