# Supplementary material for: Pomegranate Heart Rot Caused by Alternaria alternata, an Emerging Disease in Algeria
Source: J Fungi (Basel). 2026 Mar 14;12(3):209. doi: 10.3390/jof12030209 (PMC13027852; doi:10.3390/jof12030209)
Supplement: Supplementary file 1 [file jof-12-00209-s001.zip › jof-4187701-supplementary.pdf]

**Table S1**. GenBank accession numbers of ITS, EF-1α, GAPDH and OPA10-2 sequences of *Alternaria* isolates from this study and reference isolates of different host and country origins included in the phylogenetic analyses.

| Species | Isolate | Country | Host | Source | Accession numbers | | | |
|---|---|---|---|---|---|---|---|---|
| | | | | | ITS | EF-1α | GAPDH | OPA 10-2 |
| *Alternaria alternata (ex citri)* | CBS 102.47 | USA | *Citrus sinensis* | [50] | KP124304 | KP125080 | KP124161 | KP124610 |
| *A. alternata (ex-type)* | CBS 916.96 | India | *Arachis hypogaea* | [50] | AF347031 | KC584634 | AY278808 | KP124632 |
| *A. alstroemeriae* | CBS 118808 | USA | *Alstroemeria* sp. | [50] | KP124296 | KP125071 | KP124153 | KP124601 |
| *A. alternata* | AaMDc5b | Italy | *Punica granatum* | [22] | MW580754 | MW585132 | MW590513 | MW590555 |
| *A. alternata* | AaMDc5d | Italy | *P. granatum* | [22] | MW580755 | MW585133 | MW590514 | MW590556 |
| *A. alternata* | AaMMH6b | Italy | *P. granatum* | [22] | MW580756 | MW585134 | MW590515 | MW590557 |
| *A. alternata* | HRA05 | Italy | *P. granatum* | [6] | PQ593060 | PQ609992 | PQ610016 | PQ610040 |
| *A. alternata* | MNC4 | Italy | *P. granatum* | [24] | PP791896 | PP820784 | PP803581 | PP803611 |
| *A. alternata* | MNC10 | Italy | *P. granatum* | [24] | PP791902 | PP820790 | PP803587 | PP803617 |
| *A. alternata* | CBS 102595 | USA | *Citrus jambhiri* | [50] | FJ266476 | KC584666 | AY562411 | KP124636 |
| *A. alternata* | CBS 112252 | - | - | [50] | KP124340 | KP125116 | KP124194 | KP124650 |
| *A. alternata* | CBS 117.44 | Denmark | *Godetia* sp. | [50] | KP124303 | KP125079 | KP124160 | KP124609 |
| *A. alternata* | HRA09 | Italy | *P. granatum* | [6] | PQ593062 | PQ609994 | PQ610018 | PQ610042 |
| *A. alternata* | HRW59 | Italy | *P. granatum* | [6] | PQ593080 | PQ610012 | PQ610036 | PQ610060 |
| *A. alternata* | HRA10 | Italy | *P. granatum* | [6] | PQ593063 | PQ609995 | PQ610019 | PQ610043 |
| *A. alternata* | HRAW22 | Italy | *P. granatum* | [6] | PQ593077 | PQ610009 | PQ610033 | PQ610057 |
| *A. arborescens* | CBS 108.41 | - | wood | [50] | KP124394 | KP125172 | KP124246 | KP124707 |
| *A. arborescens* | CBS 109730 | USA | *Solanum lycopersicum* | [50] | KP124399 | KP125177 | KP124251 | KP124713 |
| *A. arborescens* | CBS 112749 | South Africa | *Malus domestica* | [50] | KP124401 | KP125179 | KP124253 | KP124715 |
| *A. arborescens* | AaMDc1a | Italy | *P. granatum* | [22] | MW580736 | MW580736 | MW590495 | MW590537 |
| *A. arborescens* | AaMDc1b | Italy | *P. granatum* | [22] | MW580737 | MW585151 | MW590496 | MW590538 |
| *A. arborescens* | AaMRa1 | Italy | *P. granatum* | [22] | MW580759 | MW585153 | MW590518 | MW590560 |
| *A. arborescens* | CBS 115517 | South Africa | *M. domestica* | [50] | KP124404 | KP125182 | KP124256 | KP124718 |
| *A. betae-kenyensis* | CBS 118810 | Kenya | *Beta vulgaris* var. *cicla* | [50] | KP124419 | KP125197 | KP124270 | KP124733 |
| *A. burnsii* | CBS 107.38 | India | *Cuminum cyminum* | [50] | KP124420 | JQ646305 | KP125198 | KP124734 |
| *A. eichhorniae* | CBS 119778 | Indonesia | *Eichhornia crassipes* | [50] | KP124426 | KP125205 | KP124277 | KP124741 |
| *A. jacinthicola* | CBS 878.95 | Mauritius | *Arachis hypogaea* | [50] | KP124437 | KP125216 | KP124286 | KP124753 |
| *A. longipes* | CBS 540.94 | USA | *Nicotiana tabacum* | [50] | AY278835 | KC584667 | AY278811 | KP124758 |
| *A. iridiaustralis* | CBS 118487 | Australia | *Iris* sp. | [50] | KP124436 | KP125215 | KP124285 | KP124752 |
| *A. tomato* | CBS 103.30 | - | *S. lycopersicum* | [50] | KP124445 | KP125224 | KP124294 | KP124762 |
| *A. alternata* | GA | Algeria | *P. granatum* | Present study | PX998997 | PZ013616 | PZ013598 | PZ013634 |
| *A. alternata* | GM1 | Algeria | *P. granatum* | Present study | PX998998 | PZ013617 | PZ013599 | PZ013635 |
| *A. alternata* | GM3 | Algeria | *P. granatum* | Present study | PX998999 | PZ013618 | PZ013600 | PZ013636 |
| *A. alternata* | GA5 | Algeria | *P. granatum* | Present study | PX999000 | PZ013619 | PZ013601 | PZ013637 |
| *A. alternata* | GA6 | Algeria | *P. granatum* | Present study | PX999001 | PZ013620 | PZ013602 | PZ013638 |

| | | | | | | | | |
|---|---|---|---|---|---|---|---|---|
| *A. alternata* | GA8 | Algeria | *P. granatum* | Present study | PX999002 | PZ013621 | PZ013603 | PZ013639 |
| *A. alternata* | GA9 | Algeria | *P. granatum* | Present study | PX999003 | PZ013622 | PZ013604 | PZ013640 |
| *A. alternata* | GA10 | Algeria | *P. granatum* | Present study | PX999004 | PZ013623 | PZ013605 | PZ013641 |
| *A. alternata* | GA11 | Algeria | *P. granatum* | Present study | PX999005 | PZ013624 | PZ013606 | PZ013642 |
| *A. alternata* | GA12 | Algeria | *P. granatum* | Present study | PX999006 | PZ013625 | PZ013607 | PZ013643 |
| *A. alternata* | GA13 | Algeria | *P. granatum* | Present study | PX999007 | PZ013626 | PZ013608 | PZ013644 |
| *A. alternata* | GA16 | Algeria | *P. granatum* | Present study | PX999008 | PZ013627 | PZ013609 | PZ013645 |
| *A. alternata* | GA18 | Algeria | *P. granatum* | Present study | PX999009 | PZ013628 | PZ013610 | PZ013646 |
| *A. alternata* | GA19 | Algeria | *P. granatum* | Present study | PX999010 | PZ013629 | PZ013611 | PZ013647 |
| *A. alternata* | GA20 | Algeria | *P. granatum* | Present study | PX999011 | PZ013630 | PZ013612 | PZ013648 |
| *A. alternata* | GA21 | Algeria | *P. granatum* | Present study | PX999012 | PZ013631 | PZ013613 | PZ013649 |
| *A. alternata* | GA22 | Algeria | *P. granatum* | Present study | PX999013 | PZ013632 | PZ013614 | PZ013650 |
| *A. alternata* | Tf1 | Algeria | *P. granatum* | Present study | PX999014 | PZ013633 | PZ013615 | PZ013651 |
